# Dysautonomia following Lyme disease: a key component of post-treatment Lyme disease syndrome?

**DOI:** 10.3389/fneur.2024.1344862

**Published:** 2024-02-08

**Authors:** Brittany L. Adler, Tae Chung, Peter C. Rowe, John Aucott

**Affiliations:** ^1^Division of Rheumatology, Johns Hopkins University, Baltimore, MD, United States; ^2^Department of Physical Medicine and Rehabilitation, Johns Hopkins University, Baltimore, MD, United States; ^3^Department of Pediatrics, Johns Hopkins University, Baltimore, MD, United States

**Keywords:** dysautonomia, post-treatment Lyme disease (PTLD), postural orthostatic tachycardia syndrome (POTS), *Borrelia* (Borreliella) *burgdorferi*, Lyme disease

## Abstract

Dysautonomia, or dysfunction of the autonomic nervous system (ANS), may occur following an infectious insult and can result in a variety of debilitating, widespread, and often poorly recognized symptoms. Dysautonomia is now widely accepted as a complication of COVID-19 and is an important component of Post-Acute Sequelae of COVID-19 (PASC or long COVID). PASC shares many overlapping clinical features with other infection-associated chronic illnesses including Myalgic Encephalomyelitis/Chronic Fatigue Syndrome (ME/CFS) and Post-Treatment Lyme Disease Syndrome (PTLDS), suggesting that they may share common underlying mechanisms including autonomic dysfunction. Despite the recognition of this complication of Lyme disease in the care of patients with PTLD, there has been a scarcity of research in this field and dysautonomia has not yet been established as a complication of Lyme disease in the medical literature. In this review, we discuss the evidence implicating *Borrelia burgdorferi* as a cause of dysautonomia and the related symptoms, propose potential pathogenic mechanisms given our knowledge of Lyme disease and mechanisms of PASC and ME/CFS, and discuss the diagnostic evaluation and treatments of dysautonomia. We also outline gaps in the literature and priorities for future research.

## Introduction

Dysautonomia, or dysfunction of the autonomic nervous system (ANS), often occurs following an infectious insult and can result in a variety of debilitating, widespread, and often poorly recognized symptoms. Postural orthostatic tachycardia syndrome (POTS) is one of the most common manifestations of dysautonomia, and is characterized by orthostatic and exertional intolerance due to vasomotor dysfunction, or the impaired ability to regulate blood flow (1). Patients frequently have other manifestations of dysautonomia, including but not limited to gastrointestinal dysmotility, sweating dysfunction, temperature dysregulation, sicca syndrome, and urinary and visual symptoms (2).

Over half of patients with POTS report a preceding infection (3). COVID-19 is increasingly recognized as a cause of POTS (4, 5), as well as other infections including Epstein Barr Virus (EBV) (6, 7). Lyme disease, which is caused by the tick-borne spirochete *Borrelia burgdorferi* (*B. burgdorferi*), results in chronic symptoms in ~10%−20% of patients after the infection is treated, a syndrome called Post-Treatment Lyme Disease Syndrome (PTLDS) (8). PTLDS shares many clinical similarities with other infection-associated chronic illnesses including Post-Acute Sequelae of COVID-19 (PASC), suggesting that they may share common mechanisms including dysautonomia. Although dysautonomia is reported as a complication of Lyme disease in the patient community and among some physicians, there remains a scarcity of research in this field and dysautonomia has not yet been established as a complication of Lyme disease in the medical literature. In this review, we seek to discuss the evidence implicating *B. burgdorferi* as a cause of dysautonomia and the related symptoms, propose potential pathogenic mechanisms given our knowledge of Lyme disease and mechanisms of PASC and ME/CFS, discuss the diagnostic evaluation and treatments of dysautonomia, and identify remaining gaps in the literature.

## Symptoms and clinicopathologic features of dysautonomia

Dysautonomia is a general term used to describe conditions that involve dysfunction of the ANS. The ANS, which is composed of the sympathetic, parasympathetic, and enteric nervous systems, control involuntary functions such as heart rate, blood pressure, digestion, temperature, sweating, and pupillary function. Symptoms of ANS dysregulation are diverse and include orthostatic intolerance, GI symptoms, urinary dysfunction, temperature intolerance, sicca syndrome, abnormal sweating and visual disturbances (9). Orthostatic intolerance refers to a group of symptoms that are provoked by assuming and maintaining upright posture (both sitting and standing), and many of these symptoms improve with recumbency. Once provoked, some orthostatic symptoms can persist for hours, including fatigue. Orthostatic intolerance is often associated with hemodynamic dysregulation that can lead to disabling symptoms of light-headedness, fatigue, weakness, and cognitive impairment that significantly impair quality of life.

POTS is the most common manifestation of dysautonomia. POTS affects ~1–3 million people in the United States (10–12), although this figure is likely an underestimate as dysautonomia is often overlooked, diagnostic testing is not widely available, and the COVID-19 pandemic has led to a dramatic increase in the incidence of dysautonomia that we are only now beginning to appreciate. POTS is defined as a sustained increase in heart rate >30 beats per minute in adults (or a 40 bpm increase in 12–19 year olds) within 10 min of upright posture, without a drop in blood pressure and associated with chronic orthostatic symptoms (13). Dysautonomia can also manifest as (1) neurally mediated reflex hypotension or syncope, defined as a 25-mm Hg reduction in systolic blood pressure from the baseline supine values, sustained for at least 1 min, with no associated increase in heart rate, and accompanied by symptoms of presyncope (severe weakness, lightheadedness, nausea, or diaphoresis) (14), (2) classical or delayed orthostatic hypotension (OH), defined as a drop in systolic blood pressure >20 mmHg or a drop in diastolic blood pressure >10 mmHg with standing (15, 16) within the first 3 min upright (classical OH) or after that point (delayed OH), (14) or (3) initial orthostatic hypotension, defined as a transient SBP drop ≥40 mmHg within 15 s of standing, with recovery within 45 s (17). Orthostatic intolerance confirmed by significant reductions in cerebral blood flow as measured by extracranial Doppler ultrasound can also be present even in the absence of abnormalities in heart rate and blood pressure (18).

There are several pathophysiologic subtypes of POTS which are not mutually exclusive and frequently overlap in an individual patient. These include hypovolemic POTS from reduced blood volume, hyperadrenergic POTS from increased catecholamines which can result in increased blood pressure with standing, excessive venous pooling from connective tissue laxity (19), and neuropathic POTS. Neuropathic POTS is associated with reduced intraepidermal and/or sudomotor (i.e., sweat gland) nerve fiber density on skin biopsy indicative of a small-fiber sensory or autonomic neuropathy. It is hypothesized that damage to sympathetic vasomotor nerves impairs the compensatory increase in systemic vascular resistance during orthostatic stress and/or during exercise, resulting in splanchnic and/or lower limb pooling and reduced cerebral blood flow. This results in typical POTS symptoms including dizziness, cognitive dysfunction (colloquially known as “brain fog”), and fatigue. At the same time, compensatory activation of the sympathetic nervous system in response to reduced cardiac preload results in tachycardia.

## Dysautonomia and small-fiber neuropathy often occur as a complication of certain infections

Causes of dysautonomia are heterogeneous and include infections, autoimmune disease, trauma, and neurodegenerative diseases such as multiple system atrophy (MSA). Approximately half of patients with POTS report symptoms of a preceding infection (3). Infectious causes of POTS include EBV, herpes viruses, flavivirus, enterovirus 71, retroviruses (HIV), and SARS-CoV-2 (7). Patients with infection-associated dysautonomia often report similar clinical symptoms regardless of the inciting infection. A small-fiber neuropathy has been observed across the spectrum of POTS regardless of the underlying etiology, suggesting that there may be a final common pathway that results in nerve damage (20). However, few studies have directly compared different infection-associated dysautonomia syndromes to determine if there are different clinical presentations of dysautonomia depending on the type of infection (6).

## Acute infection with *B. burgdorferi* can alter the autonomic nervous system

Left untreated, *B. burgdorferi* can infect the nervous system (neuroborreliosis) which can have a wide spectrum of presentations, the most common of which are aseptic meningitis, cranial neuropathy and radiculopathy (21, 22). Despite reports suggesting that the autonomic nervous system can also be affected, this complication of Lyme disease has received little attention. Case reports of autonomic disorders that have been reported after acute *B. burgdorferi* infection (23, 24) include intestinal pseudo-obstruction (25–27), constipation (28–30), unexplained urinary retention (28, 29), postural orthostatic tachycardia syndrome (24, 31), and reflex sympathetic dystrophy (32), which is an autonomic disorder characterized by regional sympathetic hyperactivity. Although there are other causes of urinary retention, the co-existence with enteroparesis in five of these reports implicates dysfunction of the autonomic nervous system. Another study found that 18 patients with serologically positive acute Lyme disease (IgM positive) had lower cardiac vagal tone in response to deep breathing compared to 18 healthy controls (33). In addition to autonomic symptoms, painful small-fiber neuropathy, including reduced intra-epidermal nerve fiber density (EINFD) and reduced sweat gland nerve fiber density (SGNFD), have also been documented during acute *B. burgdorferi* infection (34) and symptoms improved with antibiotics (35).

Although intriguing, these case reports are limited in number and must be interpreted with caution. In most cases, the patients had a compelling history of Lyme disease being causative given the temporal association of symptoms with an erythema migrans rash, positive Lyme serologies, and improvement with antibiotics. Several of these patients also had other symptoms that were more specific for Lyme disease such as cranial neuropathies. However, not all of the reports used strict criteria to diagnose Lyme disease. For example, several studies did not have a confirmed history of an erythema migrans rash or other objective manifestations of Lyme disease and only used serologies to diagnose Lyme disease which is not diagnostic of an active infection (31, 33). Most patients in these reports were often treated concurrently with other medications in addition to antibiotics, so it is difficult to conclude that the antibiotics alone led to symptom improvement. Furthermore, without confirmation that the patient did not experience orthostatic symptoms prior to Lyme disease and had a normal response to orthostatic stress, we cannot be sure whether dysautonomia followed the infection or preceded it. Although these studies are not without their limitations, collectively they demonstrate that untreated *B. burgdorferi* might be associated with autonomic dysregulation and small-fiber neuropathy that is potentially reversible.

Much of our understanding of the effects of *B. burgdorferi* on the autonomic nervous system come from autopsy studies in the early reports of Lyme disease as well as primate studies. Autopsy studies of patients with Lyme disease have identified lymphoplasmocellular infiltrates in the autonomic ganglia and the interstitium of the longitudinal nerves (36, 37). This is accompanied by thickening of the perineural blood vessels, which at times are surrounded by inflammatory cells. Spirochetes have never been directly visualized in the autonomic ganglia or peripheral nerves in humans, although it is likely that they are directly involved during the acute infection (38).

Studies in Rhesus monkeys have confirmed the direct effect of *B. burgdorferi* on ganglia. The sensory ganglia of macaques that were infected with *B. burgdorferi* demonstrated cellular apoptosis and necrosis of neuronal and satellite glial cells (39, 40), and the ganglia also stained positive for a lipoprotein expressed on *B. burgdorferi* suggesting that it is directly infected (39). Furthermore, rhesus DRG tissue explants exposed to live *B. burgdorferi* induced an inflammatory response and neural apoptosis in the DRG (41). These studies demonstrate that *B. burgdorferi* can directly infect the dorsal root ganglia and induce neuronal and glial cell apoptosis during acute, untreated Lyme disease. However, a limitation of this study is that it focused on the dorsal root ganglia and did not examine the sympathetic chain ganglia.

## PTLDS is a syndrome that may in some cases involve dysautonomia

Approximately 10%−20% of patients with Lyme disease develop chronic symptoms after the acute *B. burgdorferi* infection despite appropriate treatment with antibiotics (42). This syndrome is called Post-Treatment Lyme Disease Syndrome (PTLDS) and is defined as life-altering symptoms of fatigue, musculoskeletal pain, and/or cognitive difficulties that start within 6 months of the acute infection and persist for more than 6 months (8). The most common symptoms of PTLDS are fatigue, joint and muscle pain, and difficulty with concentrating and sleep, all of which are also common symptoms of dysautonomia. There is a small but notable subset of patients with PTLDS with other symptoms that are more specific to peripheral autonomic neuropathy, including difficulty with urination and nausea (43, 44), however few studies have examined this subset of patients to confirm if they have an autonomic neuropathy.

Despite the significant overlap between symptoms of PTLDS and dysautonomia, data on the role of peripheral nerve dysfunction and dysautonomia in PTLDS are lacking. In two separate case series, a total of seven patients who were treated for Lyme disease later developed new symptoms of POTS 6 months−12 years after the acute infection (45, 46). Another study of 10 patients with well-defined PTLDS who met the IDSA proposed case definition for PTLD demonstrated on tilt table test reduced cerebral blood flow velocity and either sympathetic or parasympathetic dysfunction in all participants (47). Although these reports suggest that PTLDS may be linked to dysautonomia, there are no prospective studies demonstrating a higher risk of dysautonomia in patients who previously had Lyme disease, and there are no temporal studies demonstrating a higher incidence of dysautonomia closer to the time of the acute *B. burgdorferi* infection.

Small-fiber sensory and autonomic neuropathy on skin biopsy are often found in patients with dysautonomia, and have also been linked with PTLDS. The small-fiber neuropathy provides pathologic evidence of the sensory and autonomic involvement in patients with PTLDS. In the same case series that demonstrated autonomic dysfunction in patients with PTLDS, all 10 patients had evidence of abnormal IENFD and/or SGNFD on skin biopsy (47). Reduced unmyelinated sub-basal nerve fibers in the cornea has also been described in a patient with PTLDS, and is suggestive of small-fiber neuropathy. On corneal microscopy, this patient had abundant dendritic cells, a finding which is typically seen in autoimmune diseases or chronic systemic inflammation (48).

## Potential mechanisms of infection-associated dysautonomia

**Figure 1:**
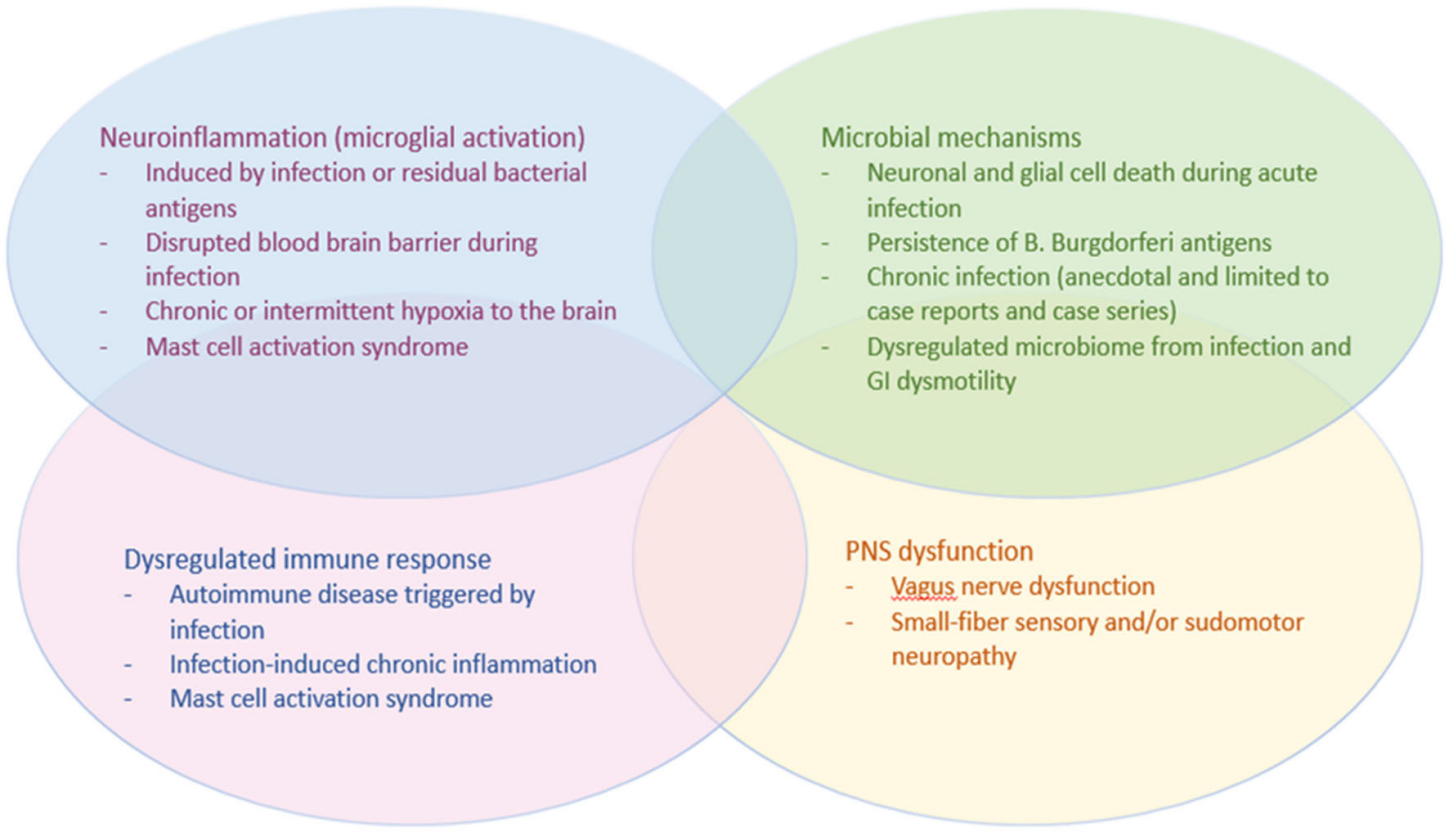
Potential mechanisms of dysautonomia in PTLDS.

The mechanisms of infection-associated dysautonomia remain unknown and research is still evolving, but studies in PTLDS and other infection-associated chronic illnesses such as PASC and Myalgic Encephalomyelitis/Chronic Fatigue Syndrome (ME/CFS) have led to several proposed hypotheses which may be applicable to dysautonomia after Lyme disease (see Figure 1). The sensory and autonomic neuropathy that is often present on skin biopsy suggests that, at least in a subset of patients, these infection-associated chronic syndromes result from a neuropathic process. The small-fiber neuropathy is often in a non-length-dependent pattern, suggesting autonomic or dorsal root ganglia as the target tissue. Although *B. burgdorferi* and SARS-CoV-2 are both known to affect the autonomic ganglia during the acute infection, there are no histopathological studies to date that have examined the ganglia or other neural tissue of patients with PASC or PTLDS. Vascular endothelial dysfunction has also been proposed as a mechanism of PASC, and more research is needed investigating the endothelium in PTLDS.

An important confounder in Lyme disease research that needs to be acknowledged when discussing mechanism is that patients can be infected with other tick-borne pathogens in addition to *B. burgdorferi*. Two percent of patients with erythema migrans have *Anaplasma phagocytophilum* and *Babesia microti* co-infections, respectively, and other infections such as Bartonella and Powassan virus have also been reported (49, 50). Some of these co-infections have been previously linked to POTS (51) and may confound human studies of PTLDS. Animal and *in vitro* studies of *B. burgdorferi* are needed to isolate the effects of *B. burgdorferi*, and human studies of Lyme disease should evaluate for tick-borne co-infections and other concurrent infections like EBV so the complete infectious profile is understood.

### Neuronal and glial cell apoptosis during the acute infection

Neuroborreliosis is well-documented, and accumulating evidence demonstrates that *B. burgdorferi* can cause neuronal and glial cell death (52). Injury to the ANS during acute infection may cause an autonomic neuropathy that is not reversible or that may take months for the nerves to regenerate. Although no studies have been performed using tissue from autonomic ganglia, *in vitro* and *in vivo* studies have demonstrated that *B. burgdorferi* can cause apoptosis of neurons and satellite glial cells in the CNS and dorsal root ganglia (41, 53–55). Neuronal cell death is thought to be caused in part by inflammatory mediators released by glial cells in response to the spirochete (56, 57). These studies suggest that *B. burgdorferi* is neurotoxic. However, pathologic studies are needed to confirm that neuronal cell death is the primary cause of chronic symptoms in PTLDS.

### Chronic *B. burgdorferi* infection in the nervous system

Persistent infection has also been invoked as a cause of PTLD-associated dysautonomia. Although previous studies in non-human primates and other animal models have demonstrated persistent *B. burgdorferi* infection after adequate antibiotic treatment (58, 59), reports of microbial persistence after antibiotic therapy in humans are anecdotal and limited (60–63). A recent study of microbial antigen persistence in late Lyme arthritis has identified *B. burgdorderi* peptidoglycan, a key component of the spirochete cell wall, in the synovial fluid of patients with antibiotic refractory late Lyme arthritis. These studies need to be confirmed by other groups and with larger cohorts of patients. Even if there is persistent infection, re-treatment with intravenous ceftriaxone followed by doxycycline does not provide dramatic or sustained improvement of symptoms in PTLDS (64). It is possible that a different antibiotic regimen is needed to treat a persistent infection and/or possible co-infections (65), as animal models have demonstrated differences in the efficacy of different antibiotics at treating *B. burgdorferi* (66). Studies of different antibiotic regimens for PTLDS are needed and should account for the presence of co-infections.

### Chronic neuroinflammation

Neuroinflammation, or inflammation of the nervous system, can arise from an infection and persist even after the infection has resolved. Imaging studies in PTLDS have identified clear evidence of microglial activation in the CNS (67), similar to what has been observed in ME/CFS myalgic encephalomyelitis (68). Research is evolving in this field to understand the mechanisms of infection-associated neuroinflammation and how it contributes to chronic symptoms (69). One proposed hypothesis is that immune system activation in response to an infection leads to the release of various cytokines and chemokines. These inflammatory mediators can cross the blood-brain barrier and activate CNS immune cells such as microglia and astrocytes, which in turn release their own inflammatory molecules and reactive oxygen species (ROS) and further propagate the immune response. It is also possible that the inflammatory response can disrupt the blood-brain barrier and allow various cytokines and chemokines to access the CNS, causing further microglial activation and neuroinflammation. Other studies suggest that non-viable spirochetal residues left-over after treatment with antibiotics may persist and retain their ability to induce inflammation and neuronal cell death (70).

Few studies have examined the relationship between autonomic dysfunction and neuroinflammation. Chronic or intermittent hypoxia in sleep apnea is associated with microglial activation in the CNS (71, 72), and is a plausible mechanism underlying neuroinflammation in patients with infection-associated dysautonomia syndromes. Studies using either extracranial Doppler ultrasound of the internal carotid and vertebral arteries or transcranial Doppler ultrasound in ME/CFS and PASC have revealed reduced cerebral perfusion in the upright position, leading to chronic positional hypoxia (18, 73–75). However, no studies have correlated cerebral perfusion with microglial activation in the infection-associated chronic syndromes. Studies in other syndromes such as fibromyalgia, which shares overlapping features with the infection-associated chronic syndromes, suggest that neuroinflammation can also involve the peripheral nervous system including the dorsal root ganglia (76). However, no studies to date have examined neuroinflammation in the autonomic ganglia of patients with infection-associated dysautonomia.

Mast cell activation is another mechanism that may contribute to neuroinflammation in patients with dysautonomia in the setting of PTLDS. POTS has been associated with mast cell activation syndrome (MCAS) (77), a poorly understood multisystem disorder of inflammation, with or without allergic phenomena or tissue growth/development anomalies (78). There is a strong bi-directional relationship between mast cells and the brain, and they serve as intermediaries between the immune system and the nervous system. Mast cells are located next to nociceptors/neurons and reside in the brain (79, 80), and when activated release neuroactive inflammatory mediators that activate microglia and can contribute to neuroinflammation (81, 82). Although MCAS is widely recognized to afflict some patients with dysautonomia, no studies to date have examined the presence of MCAS in patients with PTLDS.

### Autoimmunity triggered by the pathogen

Another hypothesis for the chronic symptoms experienced by many patients after Lyme disease is that the spirochete triggers an autoimmune response directed against a neural antigen through molecular mimicry. This hypothesis is supported by the reported lag between the acute infection and the onset of chronic symptoms, which can be 6 months or longer after the initial infection. One study found that patients with PTLDS have significantly higher anti-neural antibodies (49%) compared to healthy controls (15%) or individuals who had Lyme disease and returned to health (18.5%) (83). The specificity of these autoantibodies remains unknown, and it is not clear if these antigens are present in the central nervous system (CNS), the autonomic ganglia, the cranial nerves, or in other nervous system tissue. Antibodies which recognize lysoganglioside as well as enolase γ, an antigen present throughout the nervous system including in the ganglia, have been reported in case reports of PTLDS (84, 85). More research is needed to identify the specificities of anti-neural antibodies in PTLDS and determine if these antibodies are pathogenic.

Autoantibodies targeting specific autonomic receptors or ganglionic nicotinic acetylcholine receptors have been identified in patients with other dysautonomia syndromes and are thought to define an autoimmune subset (86). These autoantibodies include ganglionic, adrenergic, and muscarinic acetylcholine receptor antibodies (87), and correlate with autonomic symptom burden, including GI dysfunction, fatigue, muscle pain, and exercise tolerance (88). These autoantibodies are present in a subset of patients with PASC (89), although their prevalence in PTLDS remains unknown. More research is needed to validate these autoantibodies and determine their specificity for dysautonomia. It is also unclear if these autoantibodies are directly pathogenic, or whether they are a marker of autoimmunity without a specific pathophysiological effect, as the presence of various autoantibodies may simply reflect a dysregulated immune response.

Accumulating evidence suggests that a subset of patients with dysautonomia respond to the immunomodulator intravenous immunoglobulin (IVIG). There are numerous reports of patients with POTS and PASC responding to IVIG (90, 91), although data are scarce in PTLDS. The only published case report of a patient with PTLDS responding to IVIG was a patient with PTLDS and polyneuropathy who had a full recovery with subcutaneous immunoglobulin (92). Randomized clinical trials of IVIG in POTS are ongoing and are also needed in patients with PTLDS and dysautonomia.

### Vascular and endothelial damage

While it may be tempting to conclude that PTLDS is a neurologic disorder given the neurotropism of *B. burgdorferi*, research in other infection-associated chronic illnesses such as PASC have identified endothelial dysfunction as a potential driver of disease. Similar mechanisms may be applicable to other infection-associated chronic illnesses such as PTLDS. SARS-CoV-2 can directly infect endothelial cells, causing cellular damage and dysfunction that triggers an inflammatory cascade and promotes hypercoagulability, microvascular thrombosis and further endothelial dysfunction (93, 94). Fibrin amyloid microclots and platelet hyperactivation have been identified in patients with PASC and are proposed to obstruct capillaries and prevent oxygen delivery to tissues (95). More studies are needed to confirm these findings, and it remains unclear if any endothelial dysfunction results from direct viral infection or is secondary to a dysregulated immune response or an autoimmune phenomena. Similarly, *B. burgdorferi* spirochetes can adhere to and penetrate endothelial cells *in vitro*, and activate the vascular endothelium to promote transendothelial migration of neutrophils to induce an inflammatory response (96, 97). However, no studies to date have examined endothelial dysfunction in humans with PTLDS.

### Vagal nerve dysregulation

*Borrelia burgdorferi* is known to infect the cranial nerves, including the vagus nerve (CN X) (21). The vagus nerve plays a vital role in regulating numerous bodily functions and maintaining homeostasis. It is involved in the parasympathetic division of the autonomic nervous system, and is responsible for promoting rest, relaxation, and digestion, while also regulating heart rate, breathing, digestion and other involuntary processes. The vagus nerve also carries sensory information such as taste, touch and pain to the brain, and it carries motor signals from the brain to the muscles of the esophagus and digestive system.

The vagus nerve also has an important role in immune regulation. The vagus nerve transmits signals regarding peripheral inflammation to the CNS, thereby increasing brain cytokines, inducing neuro-inflammation and activating microglia (98–100), all of which have been observed in PTLDS (67, 101, 102). Moreover, the efferent pathways of the vagus nerve have a profound anti-inflammatory effect, mediated by vagus nerve-mediated cholinergic signaling (103, 104). Involvement of the vagus nerve during an acute *B. burgdorferi* infection and subsequent nerve damage may be a potential mechanism that contributes to dysautonomia and neuro-inflammation in PTLDS. Vagal nerve stimulation can ameliorate microglial activation and cytokine production (105–107), and is currently being studied as a promising therapy to improve symptoms in other infection-associated chronic syndromes including PASC and ME/CFS (108–110).

### Dysregulation of gut microbiota homeostasis

Infections may also cause dysautonomia by disrupting the gut microbiome and leading to dysregulation of the gut-brain axis. Patients with PTLDS have evidence of altered gut microbiome compared to controls with and without a history of antibiotic exposure (111). Metabolites produced by the gut microbiome, such as short-chain fatty acids, are important modulators of immune regulation, and disruption of these bacteria may contribute to neuroinflammation and autonomic dysfunction (112, 113). Conversely, GI dysmotility is common in dysautonomia and can lead to small intestinal bacterial overgrowth (SIBO) and further disrupt the gut microbiome (114, 115).

### Genetic contribution

Dysautonomia frequently runs in families, suggesting that there may be a genetic component to this syndrome which could lead to subclinical disease which is unmasked or worsened by an infection such as COVID-19 or Lyme disease. The association with joint hypermobility/Ehlers Danlos Syndrome provides further support for a genetic predisposition. Although a few pathogenic mutations have been identified in dysautonomia, such as mutations in genes encoding the norepinephrine transporter (116) or in gain of function mutations in the sodium channel (117, 118), confirmed genetic mutations are not common. Genetic polymorphisms may also play a role in the development of dysautonomia or how the disease presents clinically. Polymorphisms in endothelial nitric oxide synthase are enriched in patients with POTS (119) and polymorphisms in the beta-2 adrenoreceptor may modulate the hemodynamic profile of patients with POTS (120).

### Deconditioning

Cardiovascular deconditioning has been proposed as a mechanism underlying infection-associated dysautonomia. Over 90% of patients with POTS or orthostatic intolerance have reduced maximum oxygen uptake during exercise, which is used as a surrogate for deconditioning (121). However, there is no relationship between severity of deconditioning and cerebral blood flow reduction with tilt among ME/CSF patients, which suggests that deconditioning does not explain orthostatic intolerance (122). Furthermore, the development of orthostatic intolerance in elite athletes suggest that deconditioning is a secondary phenomenon and is not a primary driver of disease (123).

## Clinical evaluation of dysautonomia in PTLDS and potential treatments

The diagnosis of dysautonomia can be challenging, as symptoms are often nonspecific and overlap with other conditions. A comprehensive evaluation should include a detailed medical history, physical examination, and laboratory tests to rule out other medical conditions that can present similarly. Although dysautonomia has not yet been definitively established as a complication of Lyme disease, given the clinical similarities between PTLDS and other infection-associated chronic illnesses which often present with dysautonomia, we propose that patients with PTLDS should be asked about autonomic symptoms and any symptoms of dysautonomia should be rigorously evaluated with formal testing. Identifying dysautonomia in PTLDS is important because there are specific therapies that can improve autonomic symptoms, including medications that optimize hemodynamics, increase gut motility, or enhance tear and saliva production.

An autonomic evaluation includes screening for autonomic symptoms and checking orthostatic vital signs during a 10 min active or passive stand test. Symptoms of dysautonomia include orthostatic or exertional intolerance (lightheadedness or syncope, palpitations, fatigue, cognitive impairment, blurred vision or muscle weakness/pain worse with standing or activity), GI symptoms, urinary symptoms, sicca, sweating abnormalities and temperature dysregulation. Validated questionnaires such as the Composite Autonomic Symptom Score (COMPASS-31) (124), the Survey of Autonomic Symptoms (SAS) (125), or the Malmo POTS Symptom Score (126) can be used to assess autonomic symptom burden. Symptoms of dysautonomia may be further investigated with more specialized testing, including the tilt table test with or without extracranial or transcranial doppler ultrasound, heart rate variability on deep breathing, Valsalva maneuver, Quantitative Sudomotor Axon Reflex Testing (QSART) and/or thermoregulatory sweat test (TST). A skin biopsy can also be obtained to evaluate for the presence of a small-fiber sensory or sudomotor neuropathy. Table 1 highlights the most common clinically available autonomic tests.

**Table 1:** Diagnostic tests clinically available for the evaluation of dysautonomia.

**Autonomic test**	**Procedure**	**Purpose**
Head-up tilt table test or 10 min active or passive stand test	HR and BP monitoring in a supine position and head up tilt at 70 degrees on the tilt table or while standing for 10 min	To diagnose POTS: - Sustained increase in HR ≥30 bpm during head up tilt (or ≥40 bpm for ages 12–19 years) within 10 min of tilt without a drop in BP *or* HR consistently >120 bpm within 10 min of head up tilt - chronic orthostatic symptoms
		To diagnose neurally-mediated hypotension: - An abrupt drop in systolic BP of ≥25 mmHg usually with slowing of HR at the time of hypotension
Heart rate variability on deep breathing	Measures heart rate response to deep breathing	Evaluate cardiovagal reflex and/or parasympathetic cardiac innervation
Valsalva maneuver	Measures heart rate and blood pressure in response to forced expiration against resistance for up to 15 s	Assesses the body's ability to compensate for changes in the amount of blood return to the heart (preload) as an indirect measure of autonomic function Patients with POTS can have an exaggerated increase in blood pressure in response to Valsalva
Transcranial doppler ultrasound with the tilt table test	Measures changes in middle cerebral artery blood flow velocity in the upright position compared to supine	Measures the change in cerebral blood flow velocity in the brain in the upright position
Extra-cranial Doppler ultrasound with the tilt table test	Measures flow through each internal carotid and each vertebral artery	Provides a measure of total cerebral blood inflow to the brain in supine and upright positions
Quantitative Sudomotor Axon Reflex Testing (QSART)	Iontophoresis of acetylcholine, which directly stimulates sweat glands leading to evaporated sweat that can be measured by nitrogen release through use of a sudorometer	Measures postganglionic sympathetic sudomotor function
Thermoregulatory sweat test (TST)	Sweating is provoked through a warming cabinet, and the sweating pattern is assessed by color changes of alizaprin powder that is dusted over the body	Measures postganglionic sympathetic sudomotor function
Gastrointestinal motility testing	GI tests that can be obtained depending on symptoms: - Esophageal manometry - Gastric emptying study - Breath test for SIBO (small intestinal bacterial overgrowth) - Whole gut-scintigraphy - Smart pill study (whole-gut)	Measures motility of different regions of the GI tract
Skin biopsy	Quantifies the intra-epidermal and sudomotor (sweat gland) nerve fiber density in the skin	Determines if a small-fiber sensory or post-ganglionic sudomotor neuropathy is present

POTS, postural orthostatic tachycardia syndrome; HR, heart rate; BP, blood pressure; bpm, beats per minute.

There are numerous effective non-pharmacologic and pharmacologic treatments for dysautonomia, and a comprehensive review of these treatments is beyond the scope of this review. Non-pharmacologic treatments for POTS or neurogenic orthostatic hypotension include increased fluid and salt intake, use of compression stockings and an abdominal binder, and gradual increases in activity as tolerated (127). A number of pharmacologic treatments are also available for dysautonomia. For patients with hemodynamic dysregulation from POTS, heart rate can be reduced with beta-blockers or ivabradine, an inhibitor of the hyperpolarization-activated cyclic-nucleotide gate funny (*I*_f_) current (128). Fludrocortisone is a mineralocorticoid that leads to improved sodium reabsorption in the distal tubule and an early improvement in blood volume, although late effects may be secondary to improved endothelial responses to circulating vasoconstrictors. Midodrine, an α1 agonist, is used to improve vasoconstriction and thereby increase venous return to the heart; it can raise blood pressure in patients with hypotension. Pyridostigmine, a peripherally acting acetylcholinesterase inhibitor increases neurotransmission of acetylcholine to improve cardiovascular dysautonomia (16). For refractory cases, intravenous fluids can be considered for symptom management. Prokinetic agents can be used to stimulate GI motility, including serotonin receptor agonists (i.e., prucalopride), dopamine-receptor antagonists (metoclopramide, domperidone), and the acetylcholinesterase inhibitor pyridostigmine (129). Table 2 summarizes the general categories of treatments that can be considered for a patient with dysautonomia after Lyme disease.

**Table 2:** Available treatments for dysautonomia.

**Treatment approach**	**Mechanism**	**Purpose**
**Hemodynamic optimization**		
Increased fluid and sodium intake	Expands blood volume	Increases blood perfusion
Compression stockings and abdominal binder	Provides external venous compression to increase venous return	Increases blood perfusion
Physical therapy	Cardiovascular and strength training	Expands blood volume, increases cardiac and skeletal muscle mass to increase blood flow
Vasoconstrictors	Midodrine—α-1 agonist	Increases vascular tone and elevates blood pressure
	Droxidopa—Increases levels of norepinephrine in the peripheral nervous system	
	Stimulants—Increase vasoconstriction	
Cholinesterase inhibitors	Pyridostigmine—Inhibits the acetylcholinesterase (AChE) enzyme from breaking down acetylcholine (Ach)	Improves symptoms of POTS, GI dysmotility, and muscle weakness by enhancing transmission at the autonomic nerve synapses
Mineralocorticoid	Fludrocortisone—acts on the kidney to conserve sodium and water which increases plasma volume; improves response to vasoconstrictors	Increases blood perfusion
Heart-rate lowering agents	Beta-blockers—Block β-1 and/or β-2 adrenoceptors	Reduces heart rate, inhibits effects of norepinephrine and epinephrine This medication can also relax blood vessels so use caution in patients with low BP
	Ivabradine—Binds to HCN4 receptors (potassium/sodium hyperpolarization-activated cyclic nucleotide-gated channel 4)	Reduces heart rate while not affecting blood pressure
Anti-diuretics	Desmopressin (DDAVP)—synthetic version of anti-diuretic hormone increases fluid retention	Decreases standing HR without significantly affecting BP
Intravenous fluids	Increases blood volume	Increases blood perfusion
**Sympatholytics**		
Clonidine	α-2 agonist, decreases noradrenaline release both centrally and peripherally	Stabilizes HR and BP in patients with high sympathetic drive (i.e., hyper-adrenergic POTS); increases blood volume in those with orthostatic intolerance
Guanfacine and methyldopa	Decreases sympathetic nervous system tone in the brain	Stabilizes HR and BP in patients with central hyperadrenergic POTS
**Gastrointestinal motility**		
Prokinetic agents	Metoclopramide and domperidone—Dopamine D2 receptor antagonists	Amplify and coordinate GI muscular contractions
	Cisapride, prucalopride—serotonergic 5-HT_4_ receptor agonists	
	Pyridostigmine—increases acetylcholine levels	
**Stimulants**		
Stimulants	Increase dopamine and norepinephrine in the brain	Improve cognitive deficits and fatigue
Modafinil	Increases dopamine in the central nervous system	Reduces fatigue

POTS, postural orthostatic tachycardia syndrome; HR, heart rate; BP, blood pressure.

There are many clinical trials for PASC, some of which are specifically targeted at treating POTS after COVID-19. If successful, these therapies may be translated to treat dysautonomia after other infections. Many case series suggest that intravenous immunoglobulin (IVIG) is effective for PASC and POTS (90, 130), and trials of IVIG are currently underway. Efgartigimod, a neonatal Fc receptor inhibitor which leads to degradation and reduced amounts of circulating IgG, is also being studied for POTS after COVID-19 (NCT05633407). If these drugs are effective, it will strongly implicate autoimmunity as an underlying mechanism for infection-associated chronic illness. Antivirals for PASC are also being studied, which will test the hypothesis that chronic infection is an important mechanism in infection-associated chronic illnesses (131). Other potential treatments for PASC that have been proposed are beyond the scope of this review, and include the CCR5 antagonist Miraviroc along with Pravastatin (132) and neuromodulation using transcranial stimulation to improve fatigue (133). These studies in PASC, among many others, will provide insights into the mechanisms of infection-associated chronic illness that may be applied to PTLDS in the future.

## Unmet research needs

Autonomic research in PASC and ME/CFS have clearly demonstrated the important role of dysautonomia in contributing to infection-associated chronic symptoms. Because of the significant clinical similarities with PTLDS, a reasonable hypothesis is that Lyme disease, even after treatment, may be associated with dysautonomia. Although there is a sound biologic basis for this which we have reviewed and there are hints of this association in the literature, there currently are no studies clearly demonstrating this association. There are large gaps in the literature that need to be addressed to better understand the role of dysautonomia in PTLDS.

- Define the true prevalence of small-fiber neuropathy and autonomic dysfunction in PTLDS using objective autonomic testing and skin biopsies in cohorts of patients with well-defined Lyme disease.
- Understand the temporal relationship between the acute *B. burgdorferi* infection and development of small-fiber neuropathy and autonomic dysfunction.
- Expand on prior histopathological studies of involvement of *B. burgdorferi* in the autonomic nervous system using human autopsies or animal models.
- Understand the contribution of co-infections in contributing to PTLDS and dysautonomia.
- Identify distinct clinical and pathologic differences in PTLDS compared to other infection-associated chronic illnesses.

## Conclusion

Although dysautonomia is reported in case reports and case series to be a complication of treated Lyme disease, there is limited literature clearly establishing this association. Given the significant clinical similarities between post-treatment Lyme disease syndrome (PTLDS) and other dysautonomia syndromes, and the sound biological basis for the involvement of *B. burgdorferi* in the autonomic nervous system, this field of study needs more research studying this potential complication. If dysautonomia is established as a complication of Lyme disease that contributes to PTLDS, it will lead to new treatments that may improve quality of life for patients affected by this debilitating syndrome.

## Author contributions

BA: Conceptualization, Writing – original draft, Writing – review & editing. TC: Writing – review & editing. PR: Writing – review & editing. JA: Writing – review & editing.
